# The very small angle neutron scattering instrument at the National Institute of Standards and Technology

**DOI:** 10.1107/S1600576722000826

**Published:** 2022-02-27

**Authors:** John Barker, James Moyer, Steven Kline, Grethe Jensen, Jeremy Cook, Cedric Gagnon, Elizabeth Kelley, Jean Philippe Chabot, Nicholas Maliszewskyj, Chirag Parikh, Wangchun Chen, R. P. Murphy, Charles Glinka

**Affiliations:** a NIST Center for Neutron Research, National Institute of Standards and Technology, 100 Bureau Drive, Stop 6102, Gaithersbug, MD 20899, USA; b Danish Technological Institute, Gregersensvej, Taastrup DK-2630, Denmark; c University of Maryland, College Park, Maryland 20742, USA; d University of Delaware, Newark, Delaware 19716, USA

**Keywords:** small-angle neutron scattering, VSANS, instrumentation, neutron optics

## Abstract

A description and the performance of the very small angle neutron scattering diffractometer at the National Institute of Standards and Technology are presented. The measurement range of the instrument extends over three decades of momentum transfer *q* from 2 × 10^−4^ to 0.7 Å^−1^. The entire scattering angle range from 8 × 10^−5^ to π/6 rad (30°) can be measured simultaneously using three separate detector carriages on rails holding nine 2D detector arrays. Versatile choices of collimation options and neutron wavelength selection allow the *q* resolution and beam intensity to be optimized for the needs of the experiment. High *q* resolution is achieved using multiple converging-beam collimation with circular pinholes combined with refractive lenses and prisms. Relaxed vertical resolution with much higher beam intensity can be achieved with narrow slit collimation and a broad wavelength range chosen by truncating the moderator source distribution below 4 Å with a Be crystalline filter and above 8 Å with a supermirror deflector. Polarized beam measurements with full polarization analysis are also provided by a high-performance supermirror polarizer and spin flipper, capable of producing flipping ratios of over 100, along with a high-efficiency ^3^He polarization analyzer.

## Abstract 2

A description and the performance of the very small angle neutron scattering diffractometer at the National Institute of Standards and Technology are presented.

## 1. Introduction

Small-angle neutron scattering (SANS) diffractometers utilizing long flight paths, pinhole collimation, long wavelengths λ produced by cold neutron sources and 2D detectors to measure scattered intensity at small scattering angles were first developed in the 1970s in Germany at Jülich (Schelten, 1972 ▸) and are found at nearly all high-flux neutron sources today. The range of momentum transfer *q* measured by these instruments is directly related to the small-angle scattering angle θ range according to *q* ≃ 2πθ/λ. The *q* range in reciprocal space is inversely related to the microstructure feature size *d* probed as *d* ≃ 2π/*q*. A typical SANS measurement requires measuring over a large *q* range encompassing the smallest to largest features. To probe larger feature sizes requires the ability to measure to smaller scattering angles.

A relatively new class of SANS instruments, often denoted as VSANS (very small angle neutron scattering), typically push the lower limit to *q* < 0.001 Å^−1^ by combining a submillimetre-resolution 2D detector with a type of focusing optic to enhance the beam current on the sample. Other diffractometers capable of measuring to very small angles use either refractive lens focusing (Eskildsen *et al.*, 1998 ▸; Choi *et al.*, 2000 ▸), such as the SANS-U instrument at JRR-3, Tokai, Japan (Iwase *et al.*, 2011 ▸) and the Taikan instrument at J-PARC, Tokai, Japan (Iwase *et al.*, 2018 ▸), converging-beam (CB) collimation such as the TPA instrument in Saclay, France (Brûlet *et al.*, 2008 ▸; Chaboussant *et al.*, 2012 ▸) or reflective elliptical shaped mirrors such as the KWS-3 instrument in Munich, Germany (Kentzinger *et al.*, 2004 ▸; Goerigk & Varga, 2011 ▸). Reflections from horizontal mirrors are advantageous in that they correct for the beam distortion caused by gravity (Cubitt *et al.*, 2011 ▸). Spallation sources utilize broad neutron wavelength distributions via time of flight (TOF), but the broad wavelength range causes the chromatic aberration from refractive lenses and the gravity distortion to become unacceptably large. Recent developments have been made utilizing converging vertically oriented narrow slits for beam current amplification (Abbas *et al.*, 2015 ▸; Wang *et al.*, 2018 ▸). The current instrument design uses a combination of converging beams, refractive lenses and gravity-canceling prisms for beam current enhancement.

Another SANS instrument type often used to reach much smaller *q* with low instrument background is the double-crystal diffractometer (DCD). The DCD technique was first developed for X-rays by Bonse & Hart (1965 ▸), and later for neutrons by Schwahn *et al.* (1985 ▸) and Agamalian *et al.* (1997 ▸). The latter type, often referred to as ultra-SANS (USANS) instruments, utilize low-defect and highly perfect channel-cut silicon crystals for 1D collimation and have been quite successful in measuring the range *q* ≥ 3 × 10^−5^ Å^−1^. USANS instruments of this type can be found at S18 at Institut Laue–Langevin (ILL), Grenoble, France (Hainbuchner *et al.*, 2000 ▸), BT5 USANS at NIST, Gaithersburg, MD, USA (Barker *et al.*, 2005 ▸), Kookaburra at the Australian Nuclear Science and Technology Organisation (ANSTO) (Rehm *et al.*, 2013 ▸) and TOF-USANS at the Spallation Neutron Source (SNS), Oak Ridge, TN, USA (Agamalian *et al.*, 2018 ▸). Although such DCD instruments can measure over a wide *q* range, they require much longer counting times than traditional pinhole SANS instruments to obtain similar counting statistics, thus restricting the practical maximum *q* usually measured to about 0.01 Å^−1^. The 1D collimation also severely smears the data, obscuring anisotropic features easily seen using a 2D detector. Since each *q* point must be counted independently by changing the analyzer angle, such instruments have difficulty measuring a microstructure that changes with time or quantifying anisotropy in aligned samples.

**Figure 1:**
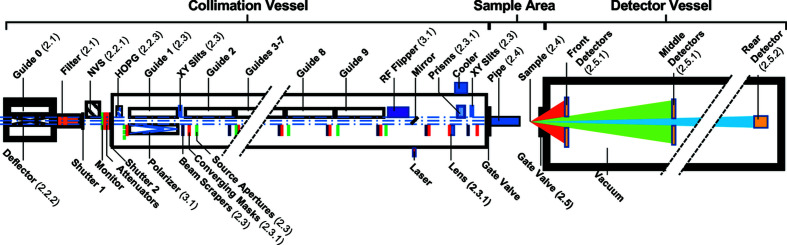
A schematic layout of the VSANS diffractometer. Numbers in parentheses () are the paper sections that describe the component.

At NIST, there are currently five SANS diffractometers. There are two 30 m-long SANS diffractometers (Glinka *et al.*, 1998 ▸) with refractive lens focusing, which cover a *q* range 1 × 10^−3^ ≤ *q* ≤ 0.6 Å^−1^. There is a pinhole 10 m SANS diffractometer accessing 2 × 10^−3^ ≤ *q* ≤ 0.6 Å^−1^ and a USANS DCD (Barker *et al.*, 2005 ▸) typically used to measure 3 × 10^−5^ ≤ *q* ≤ 3 × 10^−3^ Å^−1^. The newest addition is the VSANS diffractometer, having a range 2 × 10^−4^ ≤ *q* ≤ 0.7 Å^−1^, as described in this paper. The characteristics of the new VSANS diffractometer are summarized in Table 1 ▸, and Fig. 1 ▸ shows a schematic diagram of the instrument.

Reducing the minimum *q* limit by a factor of five with respect to the 30 m SANS instruments results in several advantages for experiments on the VSANS instrument. For example, experiments that required the broad *q* range obtained by separate measurements on the 30 m SANS and USANS instruments at different dates can now be made on a single VSANS instrument. This advantage is particularly important if the samples are unstable over a period of a few days. Consequently, the USANS measurements can now be made more quickly or with better counting statistics by removing the need to measure the typically weak scattering found at larger *q*. Anisotropic scattering samples can now be resolved to larger feature sizes, *d*, with tight 2D collimation.

A further improvement of the new instrument is the expansion of the angular range covered in a single measurement by adding additional detectors. This makes it possible to follow dynamically changing samples without time offsets in the scattering data from different *q* ranges. On the Taikan instrument at J-PARC (Iwase *et al.*, 2018 ▸), this simultaneously expanded *q* range is achieved by placing fixed tube detectors to cover a wide scattering angle range. On the new SANS instruments D33 at the ILL (Dewhurst *et al.*, 2016 ▸) and Bilby at ANSTO (Sokolova *et al.*, 2019 ▸), the expanded *q* range is achieved by arranging four 2D detectors into a picture frame, which makes a square opening in the center that allows passage of smaller scattering angle events to a second detector downstream. Neutron instruments often now rely on closely packed tube detectors using charge division to encode the neutron position along the tube’s length, thus creating a 2D detector. The advantage of tubes over other monolithic 2D gas detectors is that the detector does not need a thick pressure window or material outside the active region, thus allowing for clean transit of the inner unscattered beam. The new instrument utilizes three detector carriages: the rear carries a high-resolution 2D detector whereas the front and middle carriages use four 2D tube detectors (see the supporting information, Fig. 13) that can be moved to form a picture frame of adjustable size, thus covering the entire scattering angle range in one measurement.

In addition, the VSANS instrument incorporates several new features in a diffractometer including multiple beam collimation options and monochromator choices to optimize the instrument configuration for either high-resolution or high-flux experiments. A highly oriented pyrolytic graphite (HOPG) monochromator can be used to select a narrow wavelength distribution for a higher *q* resolution at the sacrifice of flux to measure peaks in well ordered samples. The high flux available with a wide wavelength distribution combined with the extended *q* range measured over the multiple detector carriages makes VSANS well suited for kinetics measurements and tracking temporal sample evolution. Optional polarized beam capabilities also enable the characterization of magnetic materials over a wider *q* range. These added features make the VSANS a highly versatile instrument for efficiently characterizing complex systems with hierarchical structures.

Instrument development began in 2005, and first neutron measurements were made in 2017. The instrument is supported by the National Science Foundation (NSF) through its Center for High Resolution Neutron Scattering (CHRNS) at the NIST Center for Neutron Research (NCNR). Section 2 describes the design and performance of individual components of the instrument such as wavelength selection, collimation and detector systems. Section 3 describes the polarized beam, background mitigation and scattering examples.

## 2. Diffractometer components

### 2.1. Neutron source and beam guides

The NIST reactor, hydrogen cold source and neutron guides are described in detail by Glinka *et al.* (1998 ▸), Williams & Rowe (2002 ▸) and Cook *et al.* (2015 ▸). For neutron wavelengths >3 Å, the cold source spectrum is approximately Maxwellian in distribution with an effective temperature of 30 K. The VSANS instrument is located on the NG-3 guide which is 60 mm wide by 150 mm tall and is coated with ^58^Ni to produce a critical reflection angle 1.18 times that of Ni (*m* = 1.18). The guide is straight with a length of 25 m, allowing transit of core gamma rays and fast neutrons. The distance of 25 m to the end of the guide attenuates both background source fluxes by a factor of 1.4 × 10^−5^. To protect personnel at the sample area and to reduce further the background on the instrument detectors, a filter containing a length of 20 cm of polycrystalline Be and 20 cm of randomly oriented single crystals of Bi is placed in the beam and cooled to *T* = 77 K. The Bi primarily scatters the core gamma rays, while the Be scatters the fast neutrons into surrounding shielding. The Be–Bi filter attenuates the reactor core gamma ray and fast neutron (λ < 0.3 Å) fluxes by factors of 2 × 10^−5^ and 5 × 10^−6^, respectively. To further lower the background, the instrument is rotated 0.3° to position the farthest detector out of the direct line of sight to the source.

### 2.2. Wavelength selection

The VSANS instrument has three options for selecting the mean wavelength and wavelength band delivered to the sample. Each is automated, with motors translating components into or out of the beam upstream of the collimation system.

#### 2.2.1. Neutron velocity selector

Most experiments utilize the instrument’s rotating helical neutron velocity selector (NVS), designed and built by Airbus, which provides a triangular neutron velocity distribution with mean wavelengths in the range 4.5 < λ < 20 Å depending on the rotational speed, and a fixed full width at half-maximum (FWHM) wavelength band, Δλ/λ, of 12.5%. The NVS is 250 mm in length and has 72 equally spaced 0.4 mm-thick blades with a helical twist angle of 38.5°. The inner and outer radii of the helical blades are 245 and 320 mm, respectively. The peak transmission at the mean wavelength is *T*_mon_ = 96%. The beam is located at the 3 o’clock position looking downstream and the NVS acceptance is large enough to filter the entire 6 cm wide × 15 cm tall beam.

#### 2.2.2. Supermirror deflection filter

For low resolution, Δλ/λ ≃ 44%, the NVS is translated out of the beam and a deflection filter is inserted just upstream of the Be–Bi filter. The deflector and a straight guide section are mounted side by side on a translation slide to move in or out of the beam. The deflector is a supermirror cutoff filter as described by Dewhurst (2012 ▸). The deflector is inside a rectangular guide section with an internal ‘X’-shaped supermirror as viewed from the top (see the supporting information, Fig. 12). The deflector ideally reflects neutrons having wavelength λ > 8.3 Å out of the beam, thus cutting off the long-wavelength part of the spectrum. The sharpness of the cutoff depends upon both the shape of the reflectivity curve of the supermirror with respect to the angle of incidence, and the divergence of the beam. The Be filter cuts off the λ < 4 Å short-wavelength part of the spectrum. The ‘X’ shape ensures that accepted neutrons must pass through two mirrors with reverse angles of incidence. Including both negative and positive incidence angles improves the cutoff when the beam divergence is large. The glass walls of the guide have *m* = 1 coating. The ‘X’-shaped interior substrate is 0.5 mm thick and has an *m* = 3 coating. The angle, α, between the silicon substrate and glass walls is 2.5°. For neutrons having trajectories parallel to the guide, wavelengths λ > 8.3 Å have critical angles greater than α and are reflected. The deflector and straight guide sections are both 1.43 m long and are in a helium-filled chamber.

#### 2.2.3. Pyrolytic graphite double monochromator

For the highest resolution, HOPG crystals, in a double monochromator configuration, provide a large-area beam with Δλ/λ from 0.8 to 1.0%. Two reflections are used to keep the beam direction unchanged, but the beam is offset horizontally by 8 cm. By rotating the crystals to change the angle of incidence, the wavelength can be chosen in the range 4.75 < λ < 5.5 Å. The crystals are 2 mm thick and have a nominal mosaic spread (FWHM) of 0.5°. The crystals are mounted on two parallel planes separated by 5.6 cm. Each plane has crystals that cover an area 150 mm tall by 100 mm wide. The backs of the crystals are covered with ^10^B absorber. A significant loss in beam current is incurred from the incomplete reflectivity of each reflection. The combined transmission is estimated as *T*_mon_ = 40% at λ = 4.75 Å. Note, by placing additional layers of crystals with appropriate orientation, the wavelength band and subsequent beam current can both be increased. For example, the 30 m SANS instrument in operation during the 1980s at Oak Ridge National Laboratory used six layers of HOPG crystals to produce Δλ/λ = 6% (Koehler, 1986 ▸). Under most circumstances the *q* resolution of the current instrument is dominated by angular collimation or detector spatial resolution, so that the future addition of up to five crystal layers would enhance the beam current without noticeable loss in *q* resolution.

#### 2.2.4. Be–Bi filter

**Figure 2:**
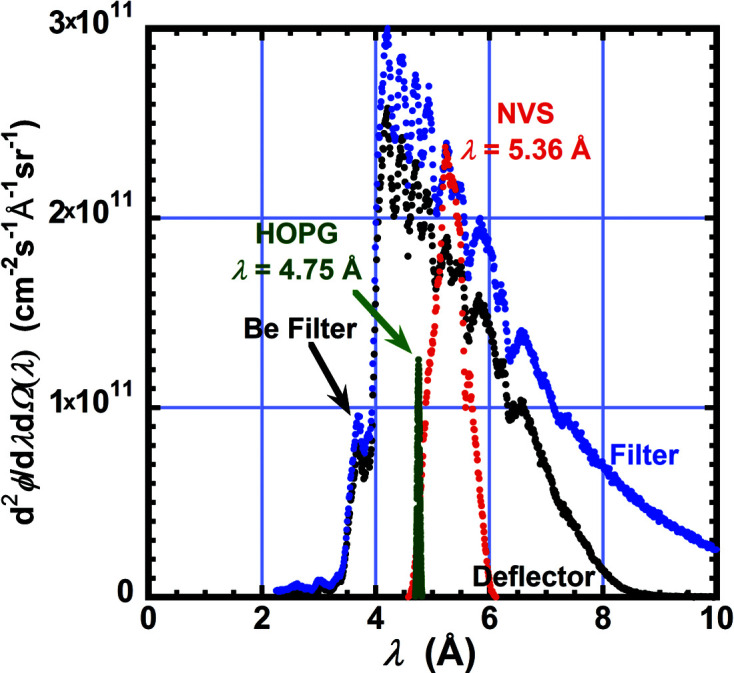
Wavelength spectra measured by neutron TOF with the filter only (blue), supermirror deflector (black), NVS (red) or HOPG (green) inserted into the beam after the Be + Bi filter. Dips in the spectra are caused by Bragg diffraction from materials in the beam, such as the Be and Bi filter and Mg and Al windows. The deflector reflects neutrons having wavelengths longer than 8 Å out of the beam. To convert to flux at the sample, multiply by the source solid angle Ω_S_ and the FWHM of the wavelength distribution Δλ for a given instrument configuration. For example, for the NVS case with all guides in (*N*_G_ = 9), λ = 5.36 Å and Δλ/λ = 0.125, the wavelength band width Δλ = 0.67 Å, the source solid angle is Ω_S_ = (π/4)×(6 cm/558 cm)^2^ = 9.08 × 10^−5^ sr and the flux at the sample is 1.5 × 10^7^ cm^−2^ s^−1^.

Another option for wavelength selection is to use all the wavelengths transmitted by the Be–Bi beam filter. This option is included in Table 2 ▸ which lists parameters that characterize each option for wavelength selection. From the wavelength distribution obtained by TOF, the mean wavelength λ and the standard deviation with respect to the mean, σ_λ_/λ, and the equivalent FWHM, Δλ/λ = (6)^1/2^σ_λ_/λ, of a triangular distribution are given. Fig. 2 ▸ plots the TOF spectrum obtained with each type of monochromator.

### 2.3. Beam collimation

The collimation system is located inside a vacuum vessel made from 11 2 m-long sections bolted together. To add flexibility and improve performance, 54 stepping motors are needed to move nearly all the components individually. A brief description of the components is given in Table 3 ▸; these include typical components found on most SANS instruments such as neutron guides, circular source apertures and beam scrapers, but also include a set of 13 converging multiple pinhole masks, stacks of lenses and prisms, two adjustable *XY* slit collimators, a polarizer guide and a radio-frequency (RF) spin flipper. For standard pinhole collimation instrument configurations, a selected number of guides *N*_G_, from 1 to 9, are inserted in the first *N*_G_ sections, and a 60 mm-diameter source aperture is inserted immediately after the last guide. The remaining sections contain beam scrapers (non-collimating apertures) to minimize background scattering from highly divergent neutrons striking unshielded components inside the vessel (Barker *et al.*, 2021 ▸). All other components are moved outside the beam in a park position. Note that, if the HOPG monochromator is used, the beam is offset by 8 cm, which requires a commensurate shift of all downstream components in the beam. For zero guides inserted, there are three choices in source aperture size of 7.5, 15 and 30 mm diameter. For CB collimation, all 12 18-hole apertures, lens and prism stacks are inserted. For narrow slit collimation, the first guide is inserted, followed by *XY* slits in sections 2 and 11 and all beam scrapers. For polarized beam operations, the double-V polarizer and RF flipper are inserted in sections 1 and 10, respectively.

#### 2.3.1. CB collimation

**Figure 3:**
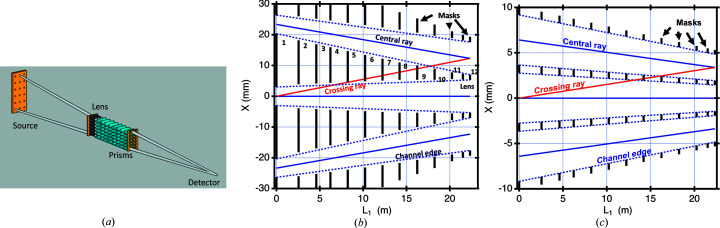
(*a*) A 3D depiction of the hybrid multiple CB 18-pinhole-collimation option available on the VSANS instrument. The beams are kept separate by a set of 13 pinhole masks roughly equally spaced within the collimation vessel (only three are shown). Between the last two masks before the sample are a stack of focusing lenses and gravity-canceling prisms for each beam. The beams converge at the beamstop in front of the high-resolution detector. (*b*) shows the partial horizontal cross section of the 13 masks used in the 18-beam CB collimation system. The beam-defining first and last apertures are 6.0 and 10.6 mm diameter, respectively. The vertical black lines denote absorbing sections of the masks. The first and last mask sizes are used for beam profile size determination. The intermediate masks block cross-talk between channels. The second to last mask fronts the lens stack. Three converging beams (blue central lines) are shown which converge at the detector located 47.2 m from the first mask. Solid lines are drawn from the center of the channels while dashed lines are drawn from the upper and lower edges. The red line shows a cross-talk ray which is blocked by masks 5, 6, 7, 8 and 9. The intermediate masks’ circular openings are oversized by 5–20% to allow for mask misalignment and changes in neutron trajectory caused by gravity. (*c*) shows an equivalent system, but without lens focusing, providing the same beam size of 12.3 mm diameter at the detector using beam-defining first and last apertures that are 5.52 and 2.91 mm diameter, respectively. To cover the equivalent sample area with the smaller sample aperture requires 10 × 21 = 210 beams. None of the intermediate mask openings are oversized. Only mask 8 blocks the potential red cross-talk ray between channels. This system would require more than the 13 masks shown to entirely eliminate cross-talk and has additional losses from potential mask misalignment and gravity distortion of the neutron path since the mask openings do not have the oversizing that is allowed by the focusing lenses.

To reach its ultimate low-*q* limit, the VSANS instrument has a hybrid collimation system consisting of 18 converging pinhole-collimated beams augmented by lens focusing and gravity canceling prisms as shown in Fig. 3 ▸(*a*). To use this configuration, 13 neutron-absorbing masks with 18 circular pinholes are translated into the beam. The individual beams converge at a beamstop at a distance 47.3 m from the first mask. By including a refractive lens stack for each beam near the sample location, we were able to gain several advantages that simplify the mask design. For conventional pinhole collimation, where the flight paths before and after the sample are nearly equal, the source aperture size is twice that of the sample aperture, and the spacing between holes in any mask is proportional to the aperture diameter. By incorporating focusing lenses, which essentially image the source aperture at the beamstop, the pinholes closer to the sample can be enlarged and more closely packed to illuminate the sample more uniformly. The smaller source pinholes can then be spaced farther apart which makes the elimination of cross-talk achievable with fewer masks and looser tolerances. Fig. 3 ▸(*b*) shows the edges of the masks for three converging beams with the current system with lens focusing, and in Fig. 3 ▸(*c*), a system without focusing but with similar sample area coverage and beam size at the detector is shown. Without lens focusing, the number of beams needed increases from 3 × 6 = 18 to 10 × 21 = 210. The individual beams are much closer together, thus requiring more than the 13 masks to eliminate cross-talk. In the VSANS system, the source pinholes are 6.0 mm in diameter, and the pinholes closest to the sample are 10.6 mm in diameter. The intermediate masks’ hole diameters are from 5 to 20% larger to accommodate the distortions in the beam path caused by gravity, misalignment during installation and distortions to vessel supports caused by strains in the vessel when evacuated.

The lens stacks consist of 14 biconcave single crystals of MgF_2_ with a radius of curvature of 12.7 mm and a diameter of 12.7 mm to focus 6.7 Å neutrons. The source-to-lens (object) distance *o* is 21.6 m and the lens-to-beamstop (image) distance *i* is 25.7 m, resulting in a magnified image of the source aperture with diameter *A*_source_ = *M* × 6 mm = 7.14 mm, where the magnification *M* = *i*/*o*. The lens stacks are followed by a stack of nine MgF_2_ prisms with a 90° apex angle to counter the effect of gravity. The path length through the prisms is much greater than that through the lens stack. Hence the prisms are cooled to 153 K to improve their transmission.

There are additional contributions to the size of the focused beams from chromatic and spherical aberrations. The additional contribution from chromatic aberration is *A*_CA_ = 2(1 + *M*)*D*_2_Δλ/λ = 5.80 mm for Δλ/λ = 12.5% and where *D*_2_ is the sample pinhole diameter. Separate calculations found that the expected contribution to the beam size from spherical aberrations is *A*_SA_ = 0.49 mm. Gravity causes the range of allowed wavelengths from 5.86 to 7.54 Å to have different parabolic paths, which stretches the beam vertically by an additional 8.3 mm at the detector, but this effect is canceled by the refraction through the prism stack (Forgcan & Cubitt, 1998 ▸). The full beam diameter is expected to be *A*_source_ + *A*_CA_ + *A*_SA_ = 13.43 mm, slightly larger than the 12 mm-diameter beamstop. Empty beam measurements found that the beamstop effectively blocked the beam, leaving only a weak parasitic halo. Future measurements are planned with a 14 mm beamstop to see if the empty background is reduced.

**Figure 4:**
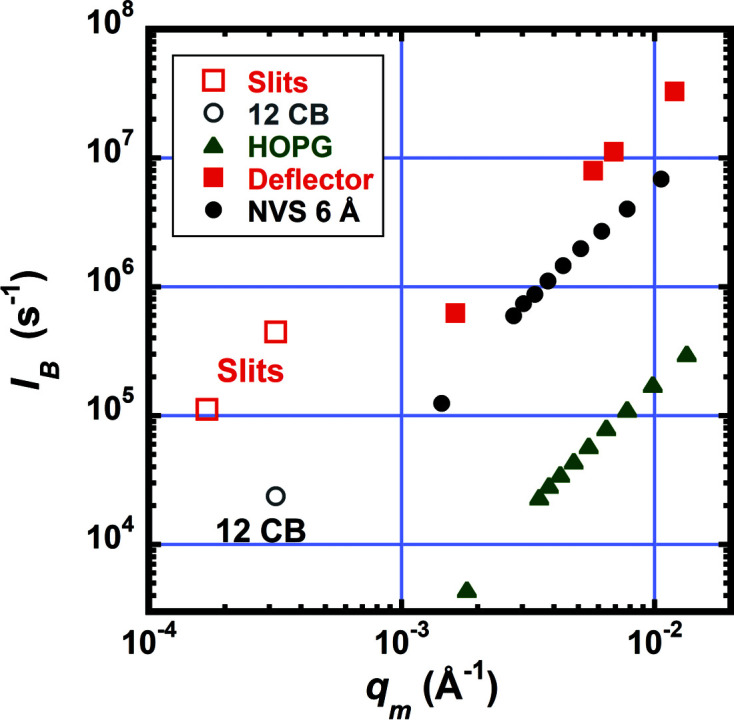
Beam current at the sample, *I*_B_, versus *q*_m_. The solid symbols represent measurements with the number of guides *N*_G_ = 0 to *N*_G_ = 9 for pinhole collimation with the NVS at λ = 6 Å (black circles), HOPG at λ = 4.75 Å (green triangles) and deflector (red squares). The open squares are the currents for two choices of slit width, 1.25 and 2.5 mm. The open circle is the current through 12 beams of the converging pinhole collimation.

The beam current *I*_B_ incident on the sample was measured for several collimation options and is plotted in Fig. 4 ▸ versus the minimum *q*, *q*_m_ = (π/λ)(*B*_S_ + Δ*r*)/*L*_2_ (*B*_S_ is the beamstop diameter or width and Δ*r* is the detector pixel size). The solid symbols represent measurements with the number of guides *N*_G_ = 0 to *N*_G_ = 9 for pinhole collimation with the NVS at λ = 6 Å (circles), HOPG at λ = 4.75 Å (triangles) and deflector (squares). The data in the open squares utilized narrow slit collimation and the open circle is for 12 converging beams. For pinhole collimation the sample aperture size was 12.7 mm diameter. For CB collimation, the sample aperture was rectangular having a 33 mm width and 44.5 mm height to pass 12 of the 18 beams. For narrow slits, the sample aperture height was 44.5 mm with widths of 1.25 and 2.5 mm when using the 6 and 12 mm-wide beamstops, respectively.

### 2.4. Sample stage

Between the vacuum vessels housing the beam collimation devices and the detector carriages is a 2 m-long open area for staging sample equipment. A rail system aligned in the beam direction allows manual movement of a permanently mounted sample stage capable of handling loads up to 900 kg over this span. The nonmagnetic sample stage has computer-controlled motors that provide rotation, vertical and horizontal motions using a high-load-capacity goniometer, lifting *z* stage and transverse slide. Thus, heavy ancillary environmentally controlled equipment such as magnets or cryostats can be easily manipulated.

To move multiple samples mounted on sample blocks into the beam, a horizontal slide is added to the stage with 1.3 m of travel, which is about three times longer than the slides on our other SANS instruments, thus greatly increasing the number of samples that can be loaded onto the slide. The most commonly used blocks are (i) a nine-position Peltier-heated or cooled block over 253 < *T* < 413 K that can independently control three temperature zones of three sample positions each; (ii) a seven-position resistive heating block having 293 < *T* < 523 K; (iii) a six-position sample holder at ambient temperature that rotates the samples (Olsson *et al.*, 2013 ▸); and (iv) a regular nine-position holder for ambient conditions. Up to three of these holders can be mounted at any given time on a long mounting plate attached to the sample slide (see the supporting information, Fig. 14). The slide can be removed to allow placement of other larger sample environments.

The large sample space was specifically designed to allow a polarized ^3^He spin analyzer to be placed downstream from even large sample environment equipment. Measurements utilizing the incident beam polarizing guide, RF spin flipper and ^3^He spin analyzer (Chen *et al.*, 2009 ▸) determine the four spin-dependent neutron scattering cross sections for magnetic samples (Krycka *et al.*, 2009 ▸; Mühlbauer *et al.*, 2019 ▸). The air path is minimized by bolting together different combinations of large- and small-diameter pipes that can be attached and evacuated with the collimation section and equipped with a sapphire window and aperture before the sample. The sapphire window has low neutron background from phonon scattering yet allows transmission of a laser beam, which facilitates aligning sample holders with the beam-defining sample aperture. To maintain vacuum in the detector vessel on the downstream side, a single-crystal silicon window, of (100) orientation and 150, 200 or 300 mm diameter, is mounted on the gate valve. By manually rotating both the end flange on the large pipe and the rectangular silicon window holder by 180°, both windows are offset by 8 cm to enable HOPG monochromator operations.

### 2.5. Post-sample flight path and detectors

Beyond the sample is the vacuum vessel housing the neutron detectors. The vessel consists of three cylindrical steel sections bolted together having a total length of 24 m and an inner diameter of 2.3 m, a rear side-hinged steel door for easy access, and a front flat plate made from nonmagnetic aluminium. The inside surface of the vessel is lined with a natural-boron-containing elastomer to absorb background-causing neutrons. To pass the beam, a 0.56 m wide × 0.38 m tall, large stainless steel gate valve is located at the front of the vessel. A 5 cm-thick aluminium plate holding a single-crystal Si(100)-oriented window seals the vacuum with the gate valve open. Rails allow three carriages to be placed independently along the beam path. Both software and hardware controls are used to avoid collisions between the carriages. The ranges in distances from the sample area defined by the upstream edge of the large gate valve to the detectors on each carriage are as follows: front 0.64–9.94 m; middle 2.65–20.67 m; rear 10.82–23.45 m. The middle and rear carriages have three beamstops each. Each beamstop can be translated horizontally over a range sufficient for alignment in the beam or removal beyond the outer edge of the closest 2D detector. Vertical adjustment is made on all beamstop supports at the same time. For a typical experiment, only one of the six beamstops is aligned to block the beam. Beamstops on the middle carriage are typically oval in shape and have widths of 50, 75 and 100 mm but are stretched vertically by 12 mm to accommodate the stretching of the beam caused by gravity. The rear carriage has a circular beamstop of 12 mm diameter, and rectangular beam stops of 6 × 300 mm and 12 × 300 mm to accommodate CB and narrow slit collimation, respectively. The vacuum in the vessel has a pressure well below 0.1 Pa before high voltage is applied to the detectors. The low pressure is required to prevent corona discharge from damaging the electronics.

#### 2.5.1. ^3^He tube 2D detectors

The front and middle carriages have four 2D detector panels each. The panels are composed of 48 stainless steel tubes spaced 8.4 mm apart, having 7.5 mm inner diameter and 0.25 mm-thick shells, and filled with 8 bar (0.8 MPa) of ^3^He. The left and right panels have vertical tube alignment with a 403 mm wide by 1040 mm tall detection area and are translated horizontally with the size of the opening between panels continuously adjustable from 5 to 360 mm. The top and bottom panels have horizontal tube alignment and are located 0.41 m behind the left and right panels to avoid collision. They can be translated independently in the vertical direction with an opening range from 5 to 320 mm, giving rise to a detection area with a height of 403 mm with a width limited by the shadow created by the opening between the left and right panels. The data along each tube are subdivided into 128 positions, corresponding to a pixel size of 8.14 mm for the long tubes in the left and right panels and 4.16 mm for the short tubes in the top and bottom panels. To avoid damage to the tube detectors, the high voltage to the tube panels is automatically turned off before any motion of the carriages, beamstops or panels. Each neutron count is processed separately and given a position and time stamp. The data are summed into separate 2D arrays for each panel for a set counting period. A sequential event file containing all the stamped events is saved on the detector computer only when requested, since the event files can be quite large.

For each tube detector the dead time, or time to process an event, was determined to be τ = 5.0 ± 0.2 µs. The data reduction software (Kline, 2006 ▸) corrects for the dead time. At very high count rates, the positional accuracy is observed to degrade to the point where the neutron position can spread evenly along the whole tube. It is recommended to keep the count rate per tube below 10 000 s^−1^ and the dead time to less than 5% to avoid spatial distortions and error in the count rate correction in the reduced scattering data.

#### 2.5.2. High-resolution 2D detector

To meet the specification that the VSANS instrument have a low-*q* limit of ≤2 × 10^−4^ Å^−1^, a 2D detector with 1–2 mm spatial resolution over an area of ∼20 × 20 cm is required. In seeking a commercial source for such a detector, various technologies were considered, including ^3^He gas detectors (Marmotti *et al.*, 2002 ▸), scintillator-based detectors (Nakamura *et al.*, 2009 ▸; Iwase *et al.*, 2012 ▸), micro-channel plate detectors (Siegmund *et al.*, 2007 ▸), semiconductor detectors (Fronk *et al.*, 2015 ▸) and others (Klein & Schmidt, 2011 ▸; Buffet *et al.*, 2005 ▸). High-pressure (0.6–1.0 MPa) ^3^He gas detectors, both multiwire proportional counters and microstrip gas counters (Clergeau *et al.*, 2001 ▸), have achieved 1–2 mm resolution in isolated cases. However, no commercial source for this technology was available during the development of the VSANS instrument. Our initial interest in a scintillation detector focused on the type of Anger camera (Riedel *et al.*, 2015 ▸) developed for the diffractometers MANDI and TOPAZ at the SNS. In this detector, light from neutron capture in the scintillator diffuses through a glass spacer before impinging on an array of multi-anode photomultipliers. The centroid of the signals from contiguous photomultipliers serves to locate the position of the neutron capture with an average resolution of <1.2 mm (FWHM). Efforts to transfer the technology for this detector to the private sector were unfortunately unsuccessful. We next turned to CCD-based scintillation detectors, which are widely used in neutron imaging applications. These are integrating detectors rather than single-event counters. Nevertheless, such detectors, utilizing low-noise CCDs and sophisticated algorithms for removing background noise, have been successfully implemented on a few diffraction and diffuse scattering instruments, for example, the CYCLOPS quasi-Laue diffractometer (Ouladdiaf *et al.*, 2011 ▸) and the D11 small-angle scattering instrument at the ILL. Both the eight CYCLOPS detectors and the D11 detectors are products of Photonic Science Ltd and use image-intensified Peltier-cooled iCCDs.

Photonic Science Ltd obtained the contract to build a customized version of their iCCD neutron detector for the VSANS instrument. The detector consists of three low-noise thermoelectrically cooled 2750 × 2200 format image-intensified CCD cameras. Each camera includes a high-sensitivity 18 mm-diameter second-generation image intensifier with adjustable gain to amplify the optical signal prior to its capture in the CCD. These cameras view separate sections of a large-area neutron scintillator via fast f0.95 close focus lenses. An integral 45° front surface mirror allows the cameras to be positioned out of the direct neutron beam. Within the cameras, the coupling between the intensifier and CCD is via a straight coherent fiber-optic that is bonded directly to the CCD.

**Figure 5:**
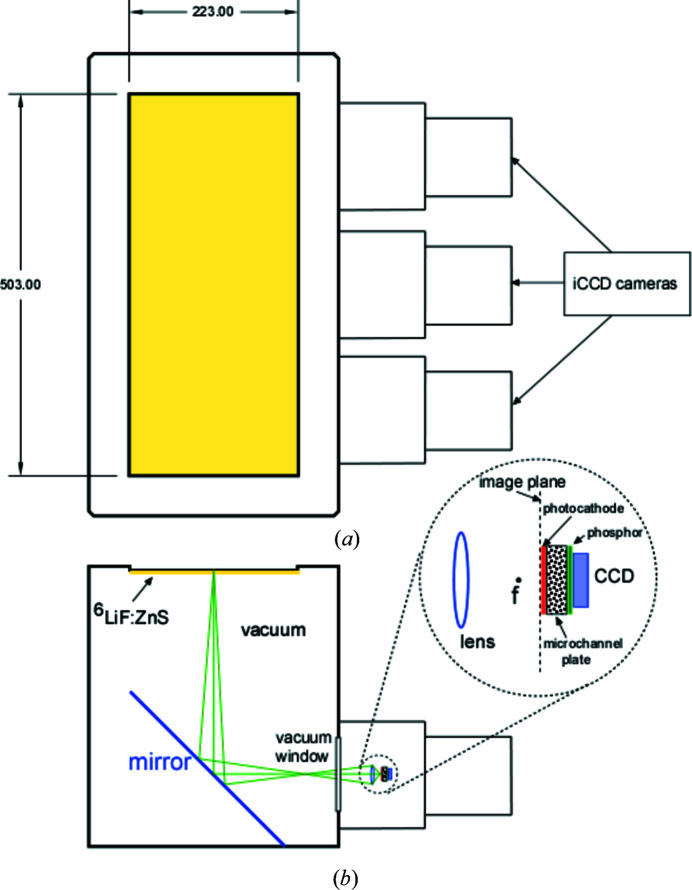
(*a*) Front view schematic of the high-resolution detector. The active region, denoted in yellow, is 223 mm wide by 503 mm tall viewed by three iCCD cameras. (*b*) Top view cross section showing the scintillator and mirror followed by vacuum-isolated light-sensitive cameras.

The scintillator is a Scintacor type ND blue, based on ^6^LiF with added ZnS:Ag. The scintillator is 0.45 mm thick with a density of ^6^Li atoms of 1.29 × 10^22^ cm^−3^, resulting in a neutron capture efficiency of 74% for 5 Å neutrons. The scintillator is bonded to a 1 mm-thick aluminium light-blocking front panel. Vent holes in the panel enable the scintillator and mirrors to be under vacuum when the detector vessel is pumped down. Light reflected from the mirrors enters the cameras via vacuum viewports. The three cameras are cooled by dual-stage Peltier thermoelectric devices with secondary water cooling. The physical and electronic characteristics of this detector are listed in Table 4 ▸. A schematic of the high-resolution detector is shown in Fig. 5 ▸.

The measured performance of the triple-iCCD detector is summarized in Table 5 ▸. The spatial resolution was determined using a narrow slit mounted on the detector. Spatial uniformity and pixel size were determined using a mask with equally spaced pinholes. Small corrections for overlap of fields of view of the individual cameras and horizontal offset were incorporated. Pixel size uniformity was within 1% with an average size of 0.32 mm pixel^−1^. Detector efficiency uniformity is within 5% over the detector and is corrected using the detector sensitivity obtained using the isotropic incoherent scattering from hydrogen. The SANS data reduction software (Kline, 2006 ▸) was modified to include separate subtraction of the initial read bias obtained from 1 s data collections with the beam turned off.

The principal shortcoming of CCD-based detectors for SANS is their considerable dark current compared with ^3^He proportional counters. Taking this into account, the dark-current specification for the Photonic Science (PS) detector was relaxed to <0.001 s^−1^ mm^−2^ (equivalent neutron counts per mm^2^ per second). This is about 400 times larger than the dark-current count rates on the NCNR’s ^3^He 2D detectors, which meant that the PS detector would be limited to measuring only the strong scattering often observed at low *q* values (<0.001 Å^−1^).

The readout from the CCD is scaled into analog-to-digital units (a.d.u.). To compare detectors, the a.d.u. corresponding to a single detected neutron had to be determined. The conversion value was obtained by comparing measurements of the beam current with the high-resolution and tube detectors. This number depends on the intensifier gain. For an intermediate gain setting of 50%, we find that 309 a.d.u. are generated per neutron capture via the tube detector. The lowest neutron-equivalent dark current that could be attained at this gain setting, after varying operating and data processing conditions over a wide range, was about 0.010 equivalent neutron counts per mm^2^ per second – about ten times above our relaxed dark-current specification. Hence only strong, steeply rising sample scattering can be measured with this detector. Since this background count rate is independent of the reactor power, the background-correction algorithm which relies on the background being proportional to the reactor power as measured by the low-efficiency beam monitor had to be modified. To further mitigate the error in background correction caused by the large dark current, most experiments are completed with the middle detector opening reduced to 50 × 50 mm, thus allowing the lower-background ^3^He detectors to measure the weaker scattering found at the outer parts of the iCCD detector.

## 3. Instrument performance measurements

### 3.1. Polarized beam

**Figure 6:**
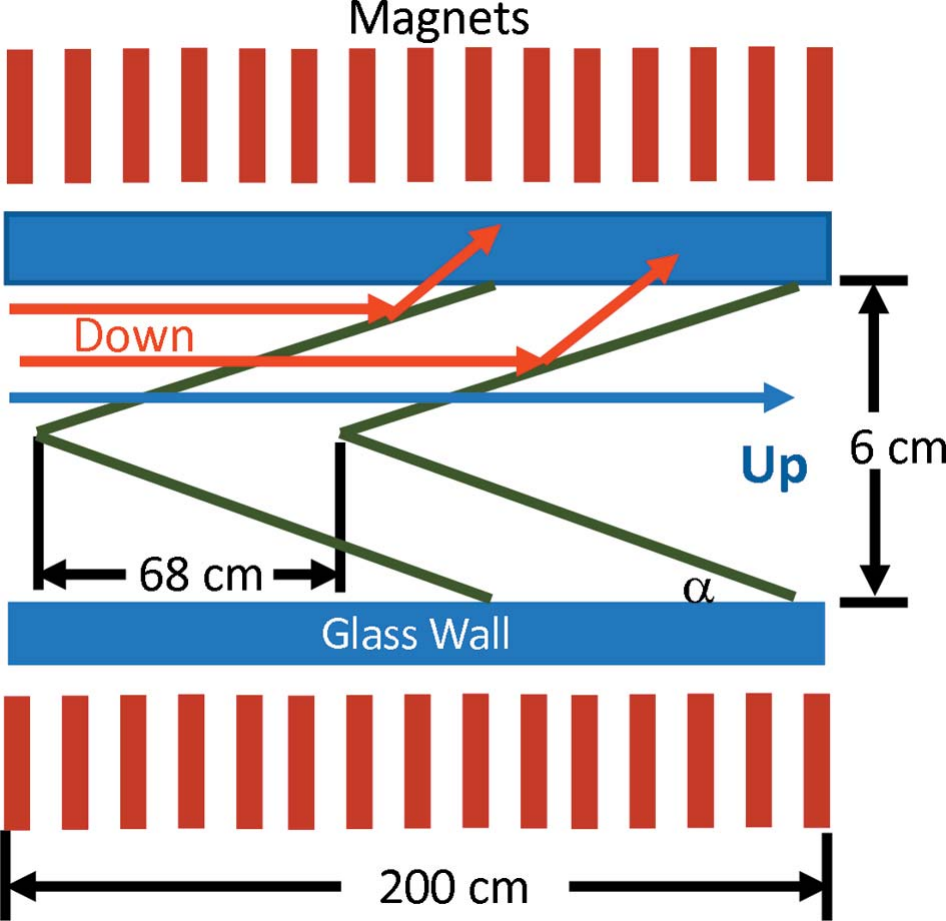
A schematic of the double-V polarizer with magnets represented in red, magnetic supermirrors in green and glass side walls in blue. The double-V silicon substrates are coated on both sides with Fe–Si supermirror (*m* = 3.5).

**Figure 7:**
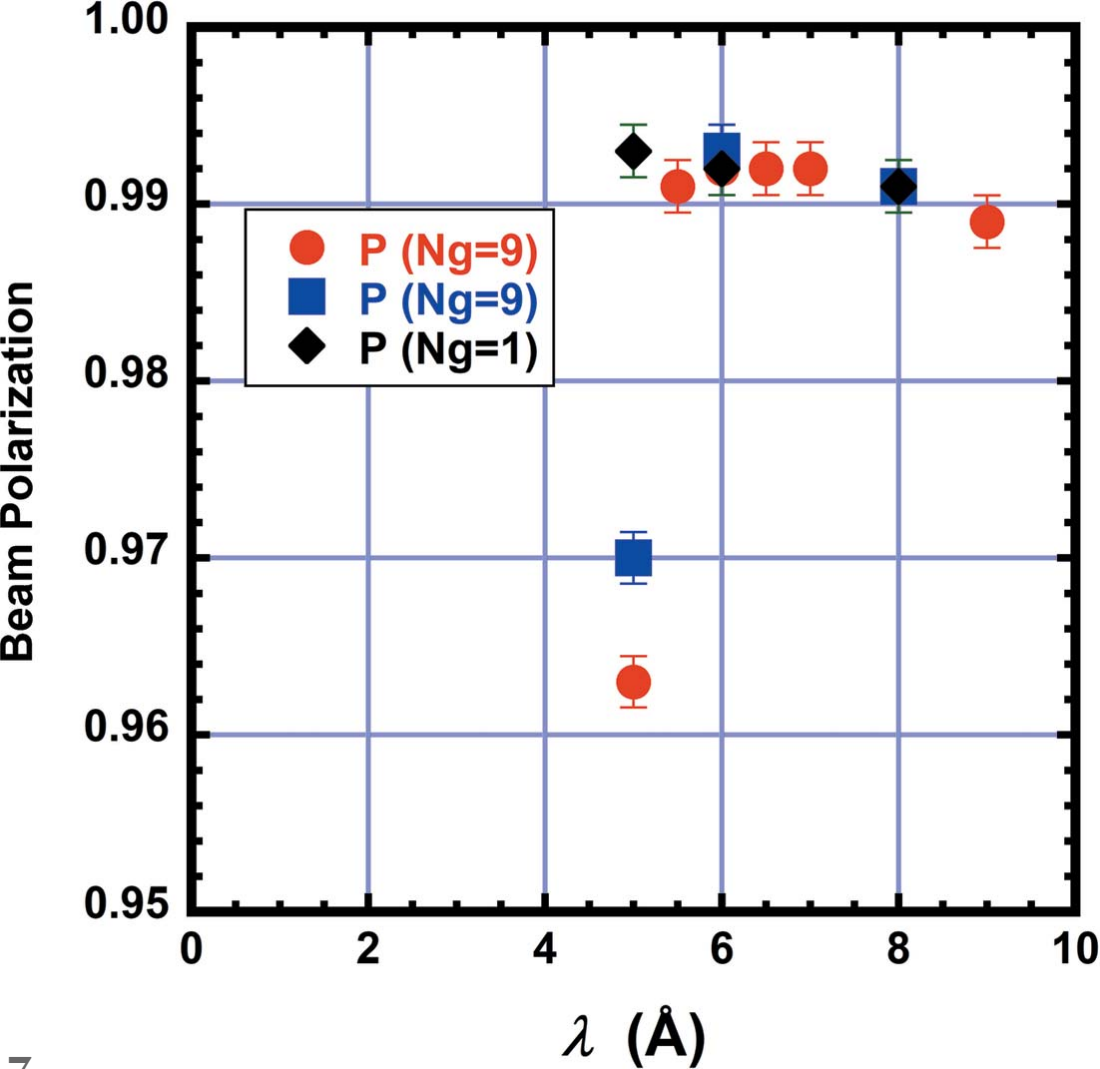
The measured polarization of the neutron beam as a function of wavelength with either one or nine neutron guides inserted. The polarization was determined from the flipping ratio, which was measured using a ^3^He spin analyzer. The red and blue data were measured in separate experiments. The larger beam divergence obtained with nine guides inserted causes a loss in beam polarization at wavelength λ = 5 Å. The error bars in all figures represent one standard deviation in uncertainty.

The 2.0 m-long polarizer guide was designed and built by Swiss Neutronics AG and is located in the first optical section. The polarizer can be automatically moved in or out of the beam. It has a double ‘V’-shaped insert of Fe–Si supermirror coating having *m* = 3.5 reflectivity on both sides of 0.3 mm-thick silicon substrates. The second coating on the back side balances the interfacial stress and thus keeps the thin wafers from warping. The incidence angle, α, for the ‘V’ is 1.396°. The offset distance between the two Vs is 68.2 cm. The inside area is 6.0 cm wide by 15.0 cm tall. A schematic of the polarizer cross section and surrounding magnetic yoke is shown in Fig. 6 ▸. Calculations by Böni *et al.* (2009 ▸) indicate such double-V polarizers should be able to produce beam polarization in excess of 99%. The polarizer sits inside a magnetic yoke to fully magnetize the Fe–Si coating. The sides of the yoke have columns of NdFeB permanent magnets, 2.5 cm wide × 5.0 cm long × 19.7 cm tall, which produce a highly uniform magnetic field of nearly 50 mT within most of the cavity, except at the far ends where the field drops to 40 mT. Fig. 7 ▸ shows the measured beam polarization at the sample location using a ^3^He analyzer. The polarization was measured with one or nine guides and as a function of wavelength. The polarization was found to be over 99% with one guide for λ ≥ 5 Å, but the polarization drops significantly for λ < 5.5 Å when the beam divergence is increased using nine guides.

A vertical guide field is used to maintain the beam polarization over the length of the instrument collimation section to the sample location. The guide field is produced by a magnetic yoke mounted outside the vacuum vessel for sections 2 through 11. The yoke produces a uniform magnetic guide field of 2.9 mT at the beam location. The top and bottom of the yoke are 0.64 cm-thick steel plates. The sides of the yoke are made from 2.5 × 5 cm steel columns with three NdFeB magnets interspersed at the top, middle and bottom of the columns. The inside of the vessel contains nonmagnetic components except for magnets found in the stepping motors and the steel screw used in the actuators. Both types of magnetic components are located far from the beam to limit field distortions.

To flip the beam polarization, an RF spin flipper is installed in the first half of the tenth optical section, as shown in the supporting information (Fig. 15). A guard aperture having a 51 mm diameter is attached to each end of the coil. The flipper is mounted on a dedicated actuator so it can be placed in or out of the beam. The external magnetic yoke is removed from this section and replaced with an internal, stepped yoke to provide a field gradient of 0.1 mT cm^−1^ along the entire length of the flipper coil with a field of 5.3 mT at the midpoint. The RF coil consists of 122 turns of 1.6 mm-diameter copper magnet wire wound around a 9 cm-diameter, 20 cm-long polyvinyl chloride (PVC) tube. The tube is sealed at its ends to a larger-diameter PVC tube so that the coil itself, and a series capacitor, occupy the air space between the tubes. The coil is part of a resonant tank circuit. The high-voltage points in the circuit are confined to the air space between the PVC tubes. Air flow between the tubes extracts about 100 W of heat produced by the tank circuit.

A pair of loosely wound excitation coils of only a few turns each spread out along the primary coil inductively couples the main coil to a pair of push–pull in-house-designed modified Hartley RF oscillators, described in detail by Chen *et al.* (2021 ▸). This circuit replaces the impedance-matched RF amplifier and waveform generator found in most RF neutron spin flipper setups. The modified Hartley oscillators act as a self-resonating bipolar power oscillator operating at a frequency of 156 kHz. The beam polarization with and without the RF flipper energized was measured to high precision using a ^3^He analyzer at λ = 5.5 Å, indicating a flipping efficiency of 0.99997 ± 3.5 × 10^−5^, with one standard deviation error estimate based upon counting statistics.

For full polarization analysis experiments, a ^3^He spin analyzer cell is placed downstream from the sample. ^3^He cells initially have polarizations as high as 85% with decay lifetimes of up to 300 h (Chen *et al.*, 2014 ▸). Both the RF spin flipper and ^3^He spin analyzer spin directions can be quickly flipped automatically from the instrument control program. Flipping ratios of up to 290 have been measured for higher-opacity ^3^He cells but with reduced beam intensity.

### 3.2. Background mitigation

**Figure 8:**
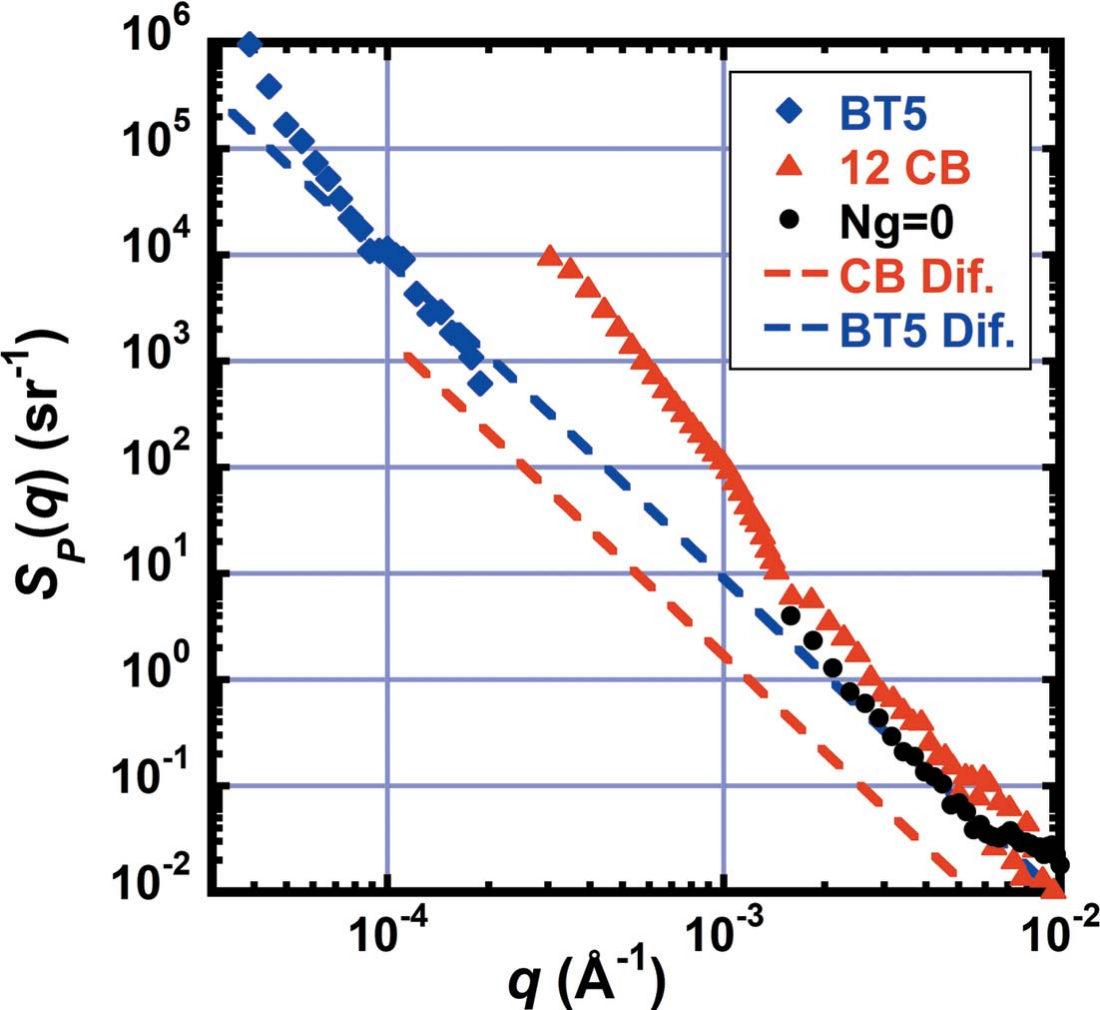
Plot of parasitic background given as a scattering probability versus *q* from the BT5 USANS instrument (blue) and two different VSANS instrument configurations: (i) 12 CB (red) and (ii) *N*_G_ = 0 pinhole collimation (black). Dashed lines are the expected diffraction from the corresponding sample apertures. The graph can be used to estimate the required scattering cross section of a sample needed to achieve a desired S/N ratio. For example, to have S/N = 1 at *q* = 0.001 Å^−1^, where the noise *S*(*q*) is ∼10^2^ sr^−1^, would require for a *d*_S_ = 0.1 cm-thick sample the macroscopic cross section dΣ/dΩ = *S*(*q*)/*d*_S_ ≃ 1000 cm^−1^ sr^−1^ at *q* = 0.001 Å^−1^.

The performance of a SANS instrument is often limited by the ratio of the scattered intensity (or signal) to the instrument background (or noise), the signal-to-noise (S/N) ratio. To achieve higher accuracy in background-corrected intensity measurements often requires increasing the S/N ratio. The noise is composed of components that are both independent of and proportional to the incident beam intensity. The noise is usually measured during an experiment by making two background measurements: (i) blocking the beam with an absorber and (ii) with no sample or empty. The difference in the background between the empty and the blocked data is termed parasitic scattering and usually scales with beam current. The parasitic background is plotted as a probability distribution in Fig. 8 ▸ for the CB and pinhole using *N*_G_ = 0 collimations and the BT5 USANS instrument. See the supporting information for details on their calculation. The background-corrected scattering intensity in absolute units (*i.e.* dΣ/dΩ) can be expressed as

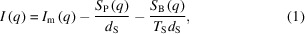

where *T*_S_ and *d*_S_ are the sample transmission and thickness, respectively, *I*(*q*) and *I*_m_(*q*) are, respectively, the absolute scattering intensity with and without background corrections and in units of cm^−1^ sr^−1^, and *S*_P_ and *S*_B_ are the background probability distributions in units of sr^−1^ with subscripts P for parasitic and B for blocked components. Note that the scattering signal is proportional to *I*(*q*), and the noise follows the same proportion as it is composed of the last two terms in equation (1). Also, the power law for slit diffraction is −3, whereas the CB data approach −4, possibly indicating some additional scattering from large features in the prism and lens material.

Beam-intensity-independent background components from the beam blocked measurement include the dark current from the detectors that can be measured with the neutron source turned off and the neutron background entering from outside the detector vessel from other instruments. The dark-current values of the ^3^He tubes and iCCD are 5 × 10^−7^ and 0.010 s^−1^ mm^−2^, respectively. The dark current from the tubes is so low that the background on the tubes is always dominated by other sources, whereas the factor of 20 000 higher dark current from the iCCD causes it to always be the dominant block background on the high-resolution detector. The tube detectors receive an additional isotropic background from neighboring instruments of 8 × 10^−6^ s^−1^ mm^−2^, a factor of 15 times that of the detector dark current, which is presumably from fast neutrons penetrating the vessel’s thermal neutron shielding. Periodic measurements indicate that this external source is quite stable during instrument operation.

Beam-intensity-dependent background components are most often dominated at large scattering angles by (i) air scattering from the sample area or (ii) phonon scattering from both the sapphire and silicon windows which are used to maintain vacuum in the collimation and detector vessels. A sample chamber is in development to allow for vacuum or backfilling with low-scattering He or Ar gas to lower the wide-angle background further.

At small angles, a parasitic halo is usually observed around the beamstop. The observed halo from our other pinhole SANS instruments usually has the same *q* ^−3^ dependence predicted by circular sample aperture diffraction (Glinka *et al.*, 2015 ▸) and is similar in strength to the *N*_G_ = 0 data in Fig. 8 ▸. The scattering from sample or guard aperture edges via refraction and reflection (Treimer *et al.*, 2002 ▸) can produce similar *q* dependence. The edge scattering from sample apertures or intermediate masks, such as beam scrapers and intermediate masks on the converging collimation system, is all mitigated by tapering the edge at 5° with the rear layer being made from a highly absorbing Cd sheet. Any scattering from the edges of source apertures is restricted to angles that lie within the beamstop by the collimation system.

### 3.3. Scattering examples

**Figure 9:**
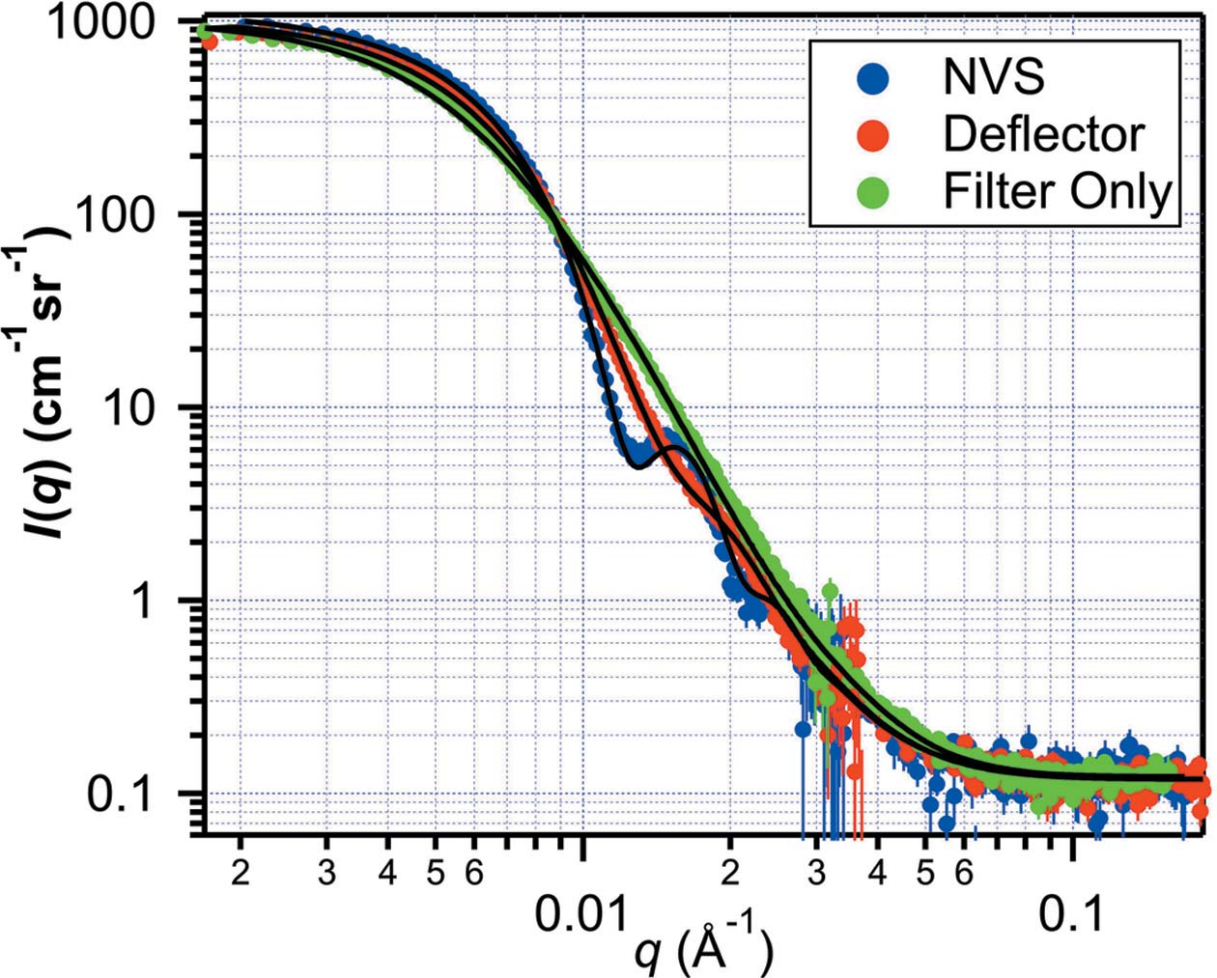
Small-angle scattering from latex spheres (PS11 source) having 370 Å nominal mean radius measured with the NVS (blue), the deflector (red) and the filter-only (green) monochromators. Solid lines are fits to a Gaussian distribution of spheres with smearing corrections. The entire *q* range was measured with one setting of the front and middle detector carriages.

Data from scattering experiments are presented here that highlight the performance of the instrument. Scattering measurements from latex spheres having a 370 Å nominal mean radius in D_2_O in a cylindrical quartz cell with 19 mm diameter and 1 mm thickness were made using *N*_G_ = 0 collimation with three different monochromator options: the NVS set at λ = 5.78 Å, the deflector and the filter. The beam was blocked on the middle carriage with the 5.0 cm oval beamstop. The middle and front carriage distances from the sample were 20 and 4 m, respectively, to cover a broad *q* range. The beamstop effectively blocked the beam for the deflector and NVS, but gravity caused the longer wavelengths to fall below the beamstop for the filter option, and part of the detector needed to be masked during data reduction for the filter-only configuration. The background-corrected count rate was enhanced by a factor of 3.8 for the deflector and 7.2 for the filter with respect to the NVS case. Fig. 9 ▸ plots the data along with fits to a Gaussian distribution of spheres, with the broader wavelength options smearing the minima in the curves.

The high flux afforded by the broader wavelength options has been used to study the kinetics in soft-matter systems, particularly when the data analysis was based on changes in the scattering invariant. Nguyen *et al.* (2019 ▸) performed a kinetics experiment on lipid vesicles that required an extended *q* range to determine the scattering invariant. They utilized the deflector monochromator option to enhance the count rate. Lee *et al.* (2019 ▸) used a similar configuration to investigate ultrasound-induced oil exchange in emulsions. The high flux and extended *q* range on VSANS have also been combined with stopped-flow mixing to enable the study of kinetic processes with a time resolution on the order of seconds (Kelley *et al.*, 2021 ▸).

**Figure 10:**
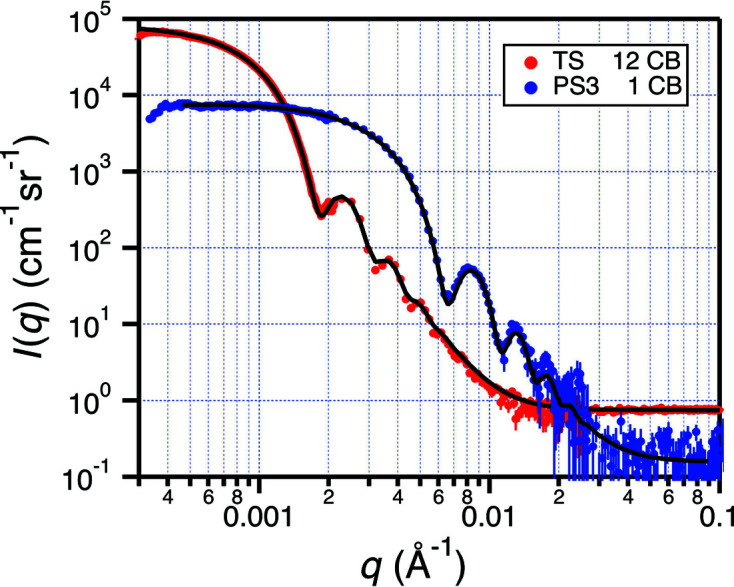
Small-angle scattering from latex spheres having 720 Å (PS3 source in blue) and 2480 Å (TS source in red) nominal mean radius *R*_0_ were measured with one and 12 converging beams, respectively. The solid lines are fits to a Gaussian distribution of spheres with smearing and multiple scattering corrections.

To test the performance at smaller *q*, the high-resolution detector was used with CB collimation on two sizes of spheres having 720 and 2480 Å nominal mean radii. Both data sets were collected with a single instrument configuration. The CB measurements were made using the NVS at λ = 6.7 Å with a 12 mm-diameter beamstop. The sample-to-detector distances for the rear, middle and front carriages were 23.4, 20 and 5 m, respectively, to cover a broad *q* range. Fig. 10 ▸ shows the corrected data and fits to a Gaussian size distribution. The fitted size distribution parameters for all latex sphere measurements are given in Table 6 ▸.

The wide *q* range accessible in a single VSANS instrument configuration has also been used to study several materials systems with hierarchical structures. The microstructure of carbon black dispersions under shear was investigated using CB collimation and the high-resolution detector by Hipp *et al.* (2021 ▸). The rheometer limited the sample width to use only one column of converging beams. The *q* range covered using all three detector carriages was 3 × 10^−4^ ≤ *q* ≤ 0.05 Å^−1^. Gilbert *et al.* (2021 ▸) used both the middle and front carriages with the NVS monochromator to extend the *q* range for kinetic measurements of the aging of micelles used in pharmaceutical formulations. Wade *et al.* (2020 ▸) made measurements with a similar instrument configuration using a rheometer which produced shear-aligned structures that exhibited strongly anisotropic scattering on the middle carriage but with additional scattering information at higher *q* found on the front carriage.

## 4. Related literature

The following references are cited in the supporting information: Jensen & Barker (2018 ▸), Rennie *et al.* (2013 ▸).

## 5. Future developments

Planning is underway to install a new liquid-deuterium neutron cold source, replacing the existing liquid-hydrogen cold source. Calculations show that the replacement will increase the source flux by a factor of two for wavelengths λ > 8 Å for all instruments viewing it. By enlarging the sapphire window and liquid cells to be able to accept a 50% taller sample aperture we can also increase the number of converging beams utilized from the current 12 to 18. Given the high dark current measured using the iCCD detector, we currently plan on replacing it with a refurbished Denex 200TN detector having an active area of 180 × 180 mm, 1 mm spatial resolution, much lower dark current and the ability to produce event mode counting for kinetics experiments. Further investigation of the causes for the higher-than-expected parasitic scattering using CB collimation will be undertaken. To lower the instrument background and to improve overlap between detectors, we plan to cover the B_4_C elastomeric shielding lining the inside of the detector vessel with Cd sheet, which has a lower albedo scattering background. Shielding of fast neutrons from neighboring instruments could significantly lower the beam-off background of the instrument. A vacuum and inert gas chamber is being designed to lower the background scattering from air and to allow measurements in a low-humidity environment. By incorporating five more layers of HOPG crystals each tilted by 0.5°, the HOPG monochromator flux can be increased by a factor of six with a concurrent increase in the wavelength spread, Δλ/λ, from 1 to 6%.

## 6. Summary

The VSANS instrument at NIST incorporates a number of new features for a SANS diffractometer. The combination of CB and lens focusing is used to enhance the beam current by allowing the use of large (33 × 44.5 mm) samples and yet minimize the beam size to 12 mm diameter on a 48 m-long collimated beam path to reach *q* = 3 × 10^−4^ Å^−1^. Narrow slit collimation with a 6 mm-wide beamstop provides enhanced beam current for measurements to *q* = 2 × 10^−4^ Å^−1^, albeit with slit smearing. New monochromator options include HOPG crystals for high resolution having wavelength spread Δλ/λ = 1%, a low-resolution supermirror deflector having Δλ/λ = 44% and a filter-only option with Δλ/λ = 80% for enhanced beam current. Full polarization analysis with flipping ratios of over 100 is available using a high-performance double-V polarizing cavity, RF spin flipper and ^3^He spin analyzer.

## Supplementary Material

Background probability distributions, sample descriptions and additional figures. DOI: 10.1107/S1600576722000826/ge5109sup1.pdf

## Figures and Tables

**Table 1:** Characteristics of the 48 m VSANS instrument

Source	*m* = 1 straight neutron guide 60 × 150 mm cross section, filter: 200 mm Be + 200 mm Bi
Monochromator	(i) HOPG, Δλ/λ = 0.8–1.0%, 4.75 < λ < 5.5 Å
	(ii) Neutron velocity selector (NVS), Δλ/λ = 12.5%, 4.5 < λ < 20.0 Å
	(iii) Deflector, Δλ/λ = 44%, λ = 5.34 Å
	(iv) Filter only, Δλ/λ = 80%, λ = 6.20 Å
Source–sample distance	Nominally 6–24 m in 2 m steps via insertion of neutron guide segments
Sample–detector distance	(i) Rear: 11.03–23.65 m
	(ii) Middle: 2.85–20.87 m
	(iii) Front: 0.84–10.14 m
Collimation	(i) Standard circular pinhole
	(ii) 18 CB with MgF_2_ lenses and prisms
	(iii) Slits; width 1–60 mm, height 1–150 mm
Sample area	2 m-long sample area with four-axis stage and vacuum pipe
*q* range	2 × 10^−4^ to 0.7 Å^−1^
Detectors	(i) Rear: ^6^LiF ZnS(Ag) scintillator with CCD, 0.85 mm resolution (FWHM), 0.21 m wide × 0.47 m tall
	(ii) Middle: 8 mm-diameter ^3^He tubes, 48 tube panels; four panels: left + right are 0.40 m wide × 1.00 m tall, top + bottom are 0.50 m wide × 0.40 m tall
	(iii) Front: same as middle (see above)
Polarization	(i) Double-V polarizer having 99% polarization
	(ii) RF spin flipper having > 99.99% flipping efficiency
	(iii) ^3^He spin analyzer

**Table 2:** The mean and standard deviation of the wavelength distributions from each type of wavelength selector obtained from TOF measurements Also included is the peak transmission, *T*_mon_, by the device with respect to the filtered beam.

Type	λ (Å)	σ_λ_/λ (%)	Δλ/λ (%)	*T*_mon_ (%)
HOPG	4.75	0.41	1.01	40
HOPG	5.5	0.31	0.77	30
NVS	6.28	5.1	12.5	96
NVS	15.9	5.1	12.5	96
Deflector	5.30	18	44	77
Be–Bi filter	6.08	33	80	100

**Table 3:** List of optical components located in the pre-sample vacuum vessel *N* is the total number of devices, *L*_1G_ is the distance from the upstream end of each device to the entrance of the large gate valve mounted on the front plate of the detector tank and Δ*L* is the length of the device.

Device	*N*	*L*_1G_ (m)	Δ*L *(m)	Opening size (mm)
Guides (*m* = 1)	9	24.5, 22.2, 20.2, 18.2, 16.2, 14.2, 12.2, 10.2, 8.2	2.0	60 × 150
Scrapers	11	22.2, 20.4, 18.5, 16.5, 14.5, 12.5, 10.4, 8.3, 6.3, 4.3, 2.5		66 × 156
Apertures	10	24.6, 21.8, 20.0, 18.0, 16.0, 14.0, 12.0, 10.0, 8.0, 6.0		7.5, 15, 30 at first and 60 diameter at rest
Converging collimators	12	24.6, 22.0, 20.0, 18.3, 16.3, 14.3, 12.3, 10.3, 8.3, 6.3, 4.3, 3.0, 2.2		18 openings varying, 6 to 10.6 diameter
Lens stack	1	3.0	0.08	18 openings, 10.6 diameter
Prism stack	1	2.8	0.23	6 openings, 56 × 11.6
*XY* slit	2	22.3, 2.4		1 to 60, 1 to 150
Polarizer	1	24.5	2.0	60 × 150
RF flipper	1	5.6	0.4	60 diameter
Shield (Pb)	3	25, 22.5, 4.6	0.1	181 × 181

**Table 4:** Physical characteristics of the Photonic Science Ltd triple-intensified (iCCD) high-resolution neutron detector

Scintillator	^6^LiF:ZnS:Ag, 0.45 mm thick from Scintacor, UK
Scintillator area	502 × 222 mm
Pixel area at scintillator	177 × 177 µm
CCD	3, Sony ICX694 (12.5 × 10.0 mm, with 6.09 million square 4.5 µm pixels)
Camera lenses	3, 25 mm diameter, f0.95
Intensifier	High-resolution (>60 line pairs mm^−1^) second-generation
Intensifier gain	Range 1000:1
Intensifier format	18 mm diameter
Intensifier photocathode	Low-noise S20 on UV transmissive substrate
Coupling to CCD	Coherent straight fiber-optic plate
Cooling	Dual-stage Peltier with secondary water cooling

**Table 5:** Measured neutron performance parameters for the VSANS high-resolution detector with intensifier set at 50% gain

Active detection area	208 × 471 mm
Spatial resolution	0.85 mm (FWHM)
Pixel size	0.32 mm
Detection uniformity	Efficiency variation <5% over entire area
Position linearity	<1% deviation from linear response
Counter saturation	8 × 10^3^ mm^−2^
Dark current	0.010 s^−1^ mm^−2^
Read offset	100 mm^−2^

**Table 6:** Gaussian size distribution fits of the mean radius *R*_0_ and the standard deviation σ_*R*_ to the latex sphere data in Figs. 9 ▸ and 10 ▸ Size information marked (Ref.) is the independent size measurement described by Hellsing *et al.* (2012 ▸) for PS11 and PS3 and by the vendor (Thermo Science) for TS. Further sample description is provided in the supporting information.

Configuration type	Particle source	*R*_0_ (Å) (Fit)	*R*_0_ (Å) (Ref.)	σ_*R*_/*R*_0_ (Fit)	σ*_R_*/*R_0_* (Ref.)
NVS	PS11	355	370	10.6%	
Deflector	PS11	356	370	12.5%	
Filter	PS11	357	370	18.4%	
1 CB	PS3	694	720	3.8%	2%
12 CB	TS	2473	2480	6.9%	1.7%
