# Supplementary material for: The very small angle neutron scattering instrument at the National Institute of Standards and Technology
Source: J Appl Crystallogr. 2022 Feb 27;55(Pt 2):271–83. doi: 10.1107/S1600576722000826 (PMC8985601; doi:10.1107/S1600576722000826)
Supplement: Supplementary file 1 [file j-55-00271-sup1.pdf]

JOURNAL OF APPLIED CRYSTALLOGRAPHY

**Volume 55 (2022)**

**Supporting information for article:**

# The very small angle neutron scattering instrument at the National Institute of Standards and Technology

**John Barker, James Moyer, Steven Kline, Grethe Jensen, Jeremy Cook, Cedric Gagnon, Elizabeth Kelley, Jean Philippe Chabot, Nicholas Maliszewskyj, Chirag Parikh, Wangchun Chen, R. P. Murphy and Charles Glinka**

## I. Instrument background expressed as Scattering Probability (see section 3.2)

Here we describe the method used to convert measured scattering background in arbitrary units into a scattering probability distribution as used in Fig. 8. The advantage of using the scattering probability is that background data can be compared directly between different instrument configurations. Scattering data from the 2D detectors is radially averaged and is corrected for instrument background by

$$i_C(q) = i_S(q) - T_s i_E(q) - (1 - T_S) i_B(q) = i_S(q) - T_s i_P(q) - i_B(q) \tag{2}$$

where subscripts C, S, E and B represent the corrected, sample, empty and blocked runs, respectively, $q$ depends upon the mean scattering angle in the annuli and

$$i_P(q) = i_E(q) - i_B(q) \tag{3}$$

where P represents parasitic background. The above values are usually normalized to a beam monitor count, but for simplicity, we here assume normalization simply by counting time, so the raw $i$ functions all have units of $s^{-1}$. The corrected data is placed in absolute units by

$$I(q) = \frac{i_C(q)}{I_B d_S T_S \Delta\Omega(q)} \tag{4}$$

where $I_B$ is the beam current incident on the sample and $\Delta\Omega(q)$ is the solid angle in each annuli of $q$. The background measurements can be converted to a scattering probability distribution over solid angle such that we reproduce equation (1)

$$I(q) = I_m(q) - \frac{S_P(q)}{d_S} - \frac{S_B(q)}{d_S T_S} \tag{1}$$

where $I_m(q)$ is calculated by equation (4) by substituting $i_S(q)$ for $i_C(q)$. The background probability distributions are

$$S_P(q) = \frac{i_P(q)}{I_B \Delta\Omega(q)}$$

$$S_B(q) = \frac{i_B(q)}{I_B \Delta\Omega(q)} \tag{5}$$

The background probability distributions $S$ can then be used directly to estimate the background or noise level and compared to model calculations of the absolute intensity $I(q)$ used to estimate the signal of an unmeasured sample.

Note the direct beam profile behind the beam stop will integrate over solid angle to near unity because the parasitic component in the halo beyond the beam stop is normally quite small. For example, the CB collimation has 0.6% beyond the beam stop. If the beam profile has the shape of a cone with an outer extent $q_B$, the cone profile expressed as a scattering probability is

$$S_P(q) = S_P(0)\left[1 - \frac{q}{q_B}\right]$$

where

$$S_P(0) = \frac{12\pi}{q_B^2\lambda^2} \tag{6}$$

As collimation is tightened thus decreasing $q_B$ , the direct beam component will continue to strengthen in amplitude independently from the actual parasitic background of the instrument. Since the diffraction component of the background follows $S_P(q) \sim q^{-3}$, it follows that $S_P(q_B)/S_P(0) \sim q_B^{-1}$. Some publications (Barker *et al*. 2005) have utilized the above ratio measured at twice the fwhm of the beam profile or $2q_B$ as a measure of instrument performance. It can be shown that if the parasitic background obeys circular aperture diffraction and with a cone beam profile that

$$\frac{S_P(2q_B)}{S_P(0)} = \frac{1}{24\pi R_2 q_B} \tag{7}$$

Fig 11 shows the data from Fig. 8 where the direct beam profiles have been added. The measured parasitic component for CB collimation is about one order of magnitude higher then the aperture diffraction prediction.

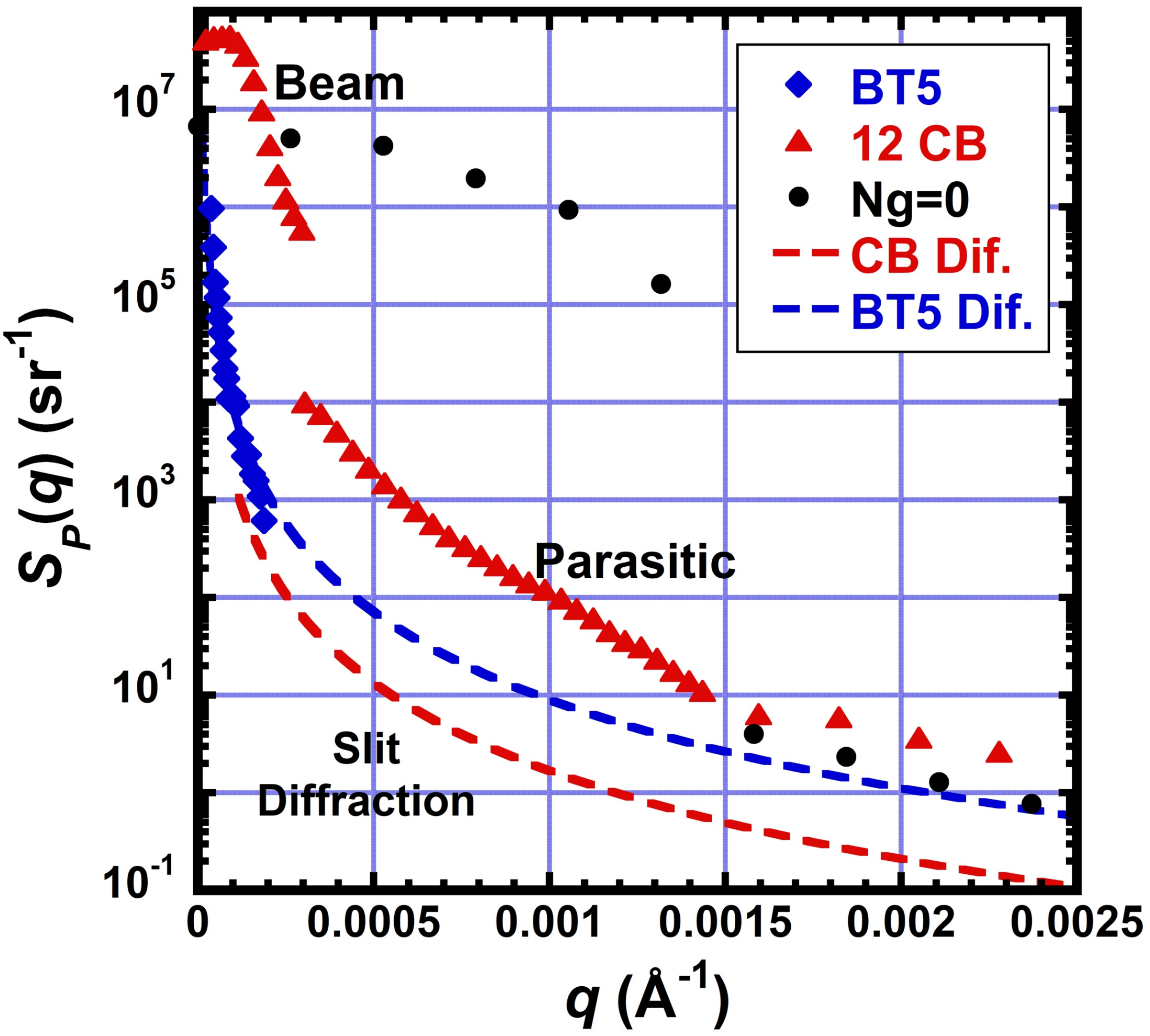


Figure 11. Parasitic background for the BT5 USANS instrument (blue), and two different VSANS instrument configurations: i) twelve CB (red) and ii) $N_G = 0$ pinhole collimation (black). Dashed lines are the expected diffraction from the corresponding sample apertures. Same data as in Fig. 8, but with the inclusion of the direct beam with the beam stop removed for both VSANS configurations.

**II. Latex Particle Solution Description (see section 3.3)**

Scattering from three different size polystyrene latex particles is shown in Fig. 9 and 10. Electrolyte is added to reduce the correlation between particles. PS11 and PS3 particles are in 100 % $D_2O$ and particle manufacture and characterization is described in Hellsing *et al*. 2012 and Rennie *et al*. 2013. The TS particles were obtained from Thermo Scientific (TS) with supplied size distribution characterization. To minimize sedimentation, the TS sample was suspended in 45 volume % $D_2O$ and 55 % $H_2O$ to make the solution have similar mass density as the latex particles. Table 7 lists the known size information. The scattering power $\tau$ for multiple scattering correction is estimated from equation (14) in Jensen & Barker (2018) by using a Guinier fit to obtain $I(0)$, sample thickness $d_S$ and $<\lambda^2>$ weighted mean neutron wavelength from scattering measurement. Multiple scattering in data fitting is approximated by using only double scattering for the mean particle size.

Table 7. Mean particle radius $R_0$ , standard deviation in radius $\sigma_R$ and scattering power $\tau$ for the three different latex particle samples.

| Source code | $R_0$ | $\sigma_R/R_0$ | $d_S$ | $\lambda$ | $\tau$ |
|---|---|---|---|---|---|
| PS11 | 370 Å | - | 0.1 cm | 5.78 Å | 0.009 |
| PS3 | 720 Å | 2% | 0.2 cm | 6.7 Å | 0.046 |
| TS | 2480 Å | 1.7% | 0.2 cm | 6.7 Å | 0.046 |

**III. Supporting Figures**

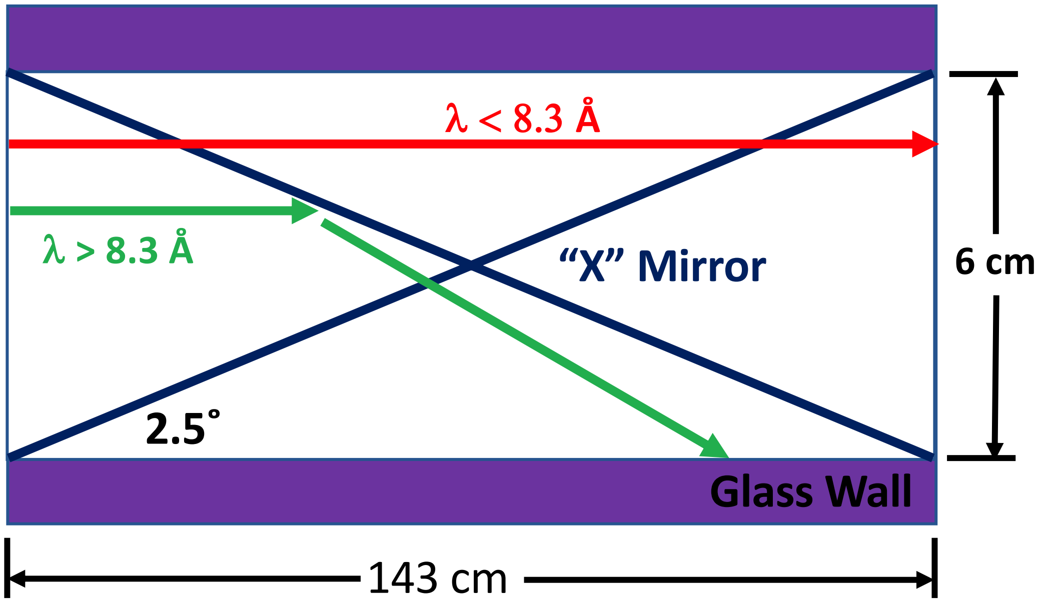


Figure 12. Diagram of the "X" deflector. (see section 2.2.2). Horizontal section taken from mid-height. Red rays ($\lambda < 8.3$ Å) are transmitted while green rays ($\lambda > 8.3$ Å) are reflected out of the beam. The deflector is housed in a 1.43 m long guide having m =1 coating on the four inner walls. The "X" deflector is composed of two mirrors having supermirror coating of m = 3 set at an angle of 2.5˚ from the guide walls that cross the 6 cm width of the guide.

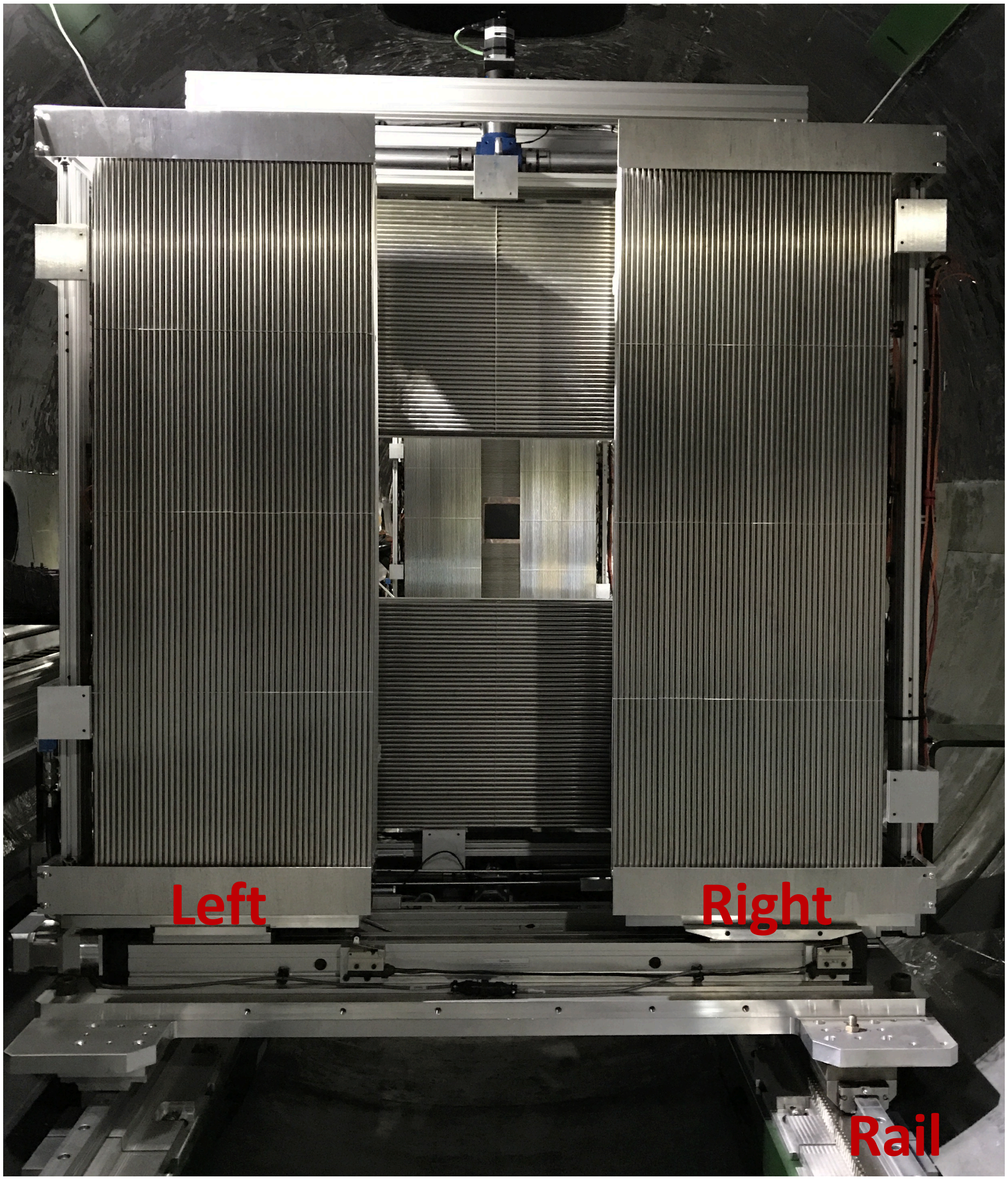


Figure 13. Shows a picture of all eight tube detector panels. (see section 2.5.1). The four panels on the front carriage form a picture frame around the four panels on the middle carriage. The middle carriage forms a second picture frame around a central region that passes onto the rear detector.

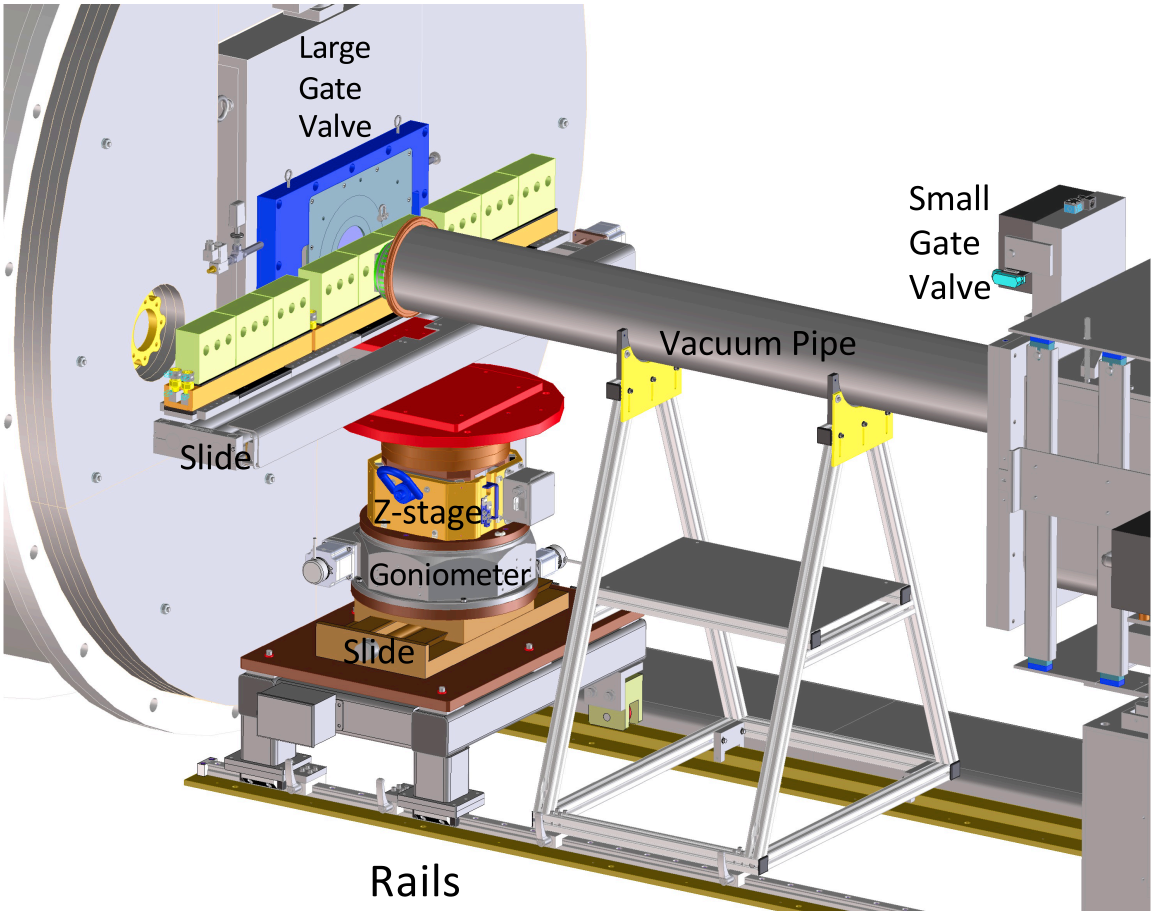


Figure 14. The VSANS instrument sample area in a typical configuration with three nine-position temperature-controlled sample holders on a translation slide. (see section 2.4).

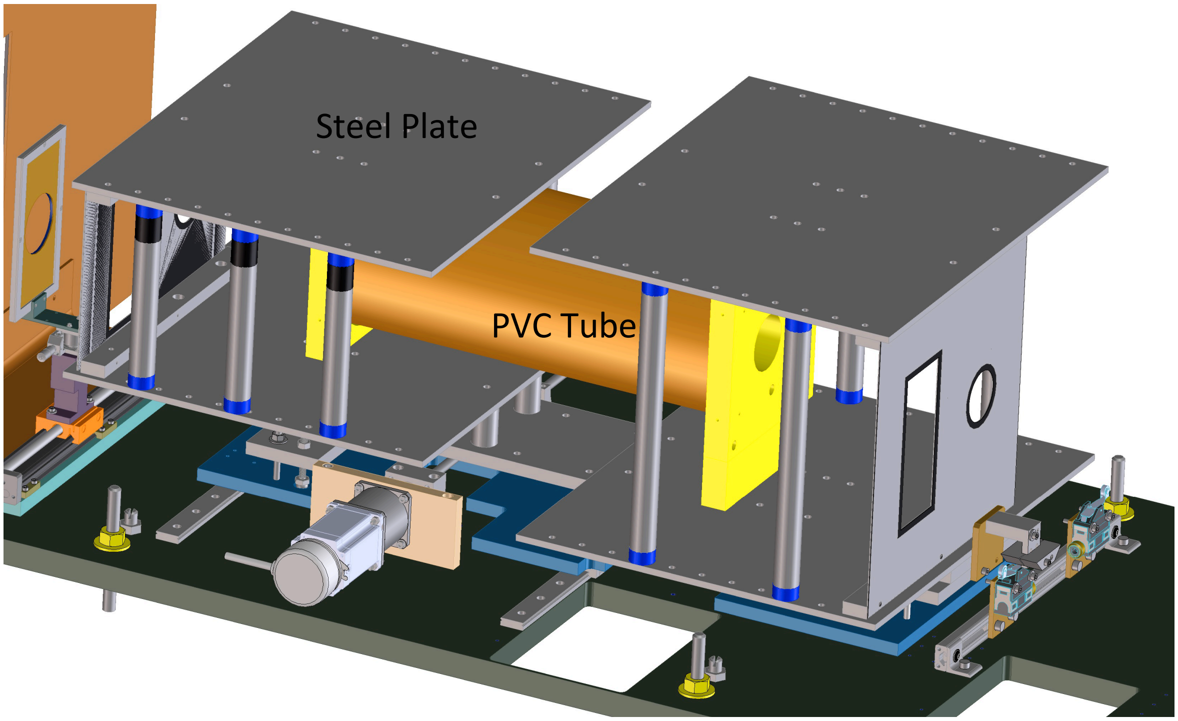


Figure 15. A 3D view of components in the 10th optical section containing the RF spin flipper. (see section 3.1). The gray steel plates are supported by gray steel columns with magnets (shown in blue). The coil is mounted in air between two concentric PVC tubes shown in orange. The stepped plates provide a static magnetic field with a linear gradient.

**Supporting References**

Jensen, G. V. & Barker, J. G. (2018). *J. Appl. Cryst.* **51**, 1455-1466.

Rennie, A. R., Hellsing, M. S., Wood, K., Gilbert, E. P., Porcar, L., Schweins, R., Dewhurst, C. D., Lindner, P., Heenan, R. K., Rogers, S. E., Butler, P. D., Krzywon, J. R., Ghosh, R. E., Jackson, A. J. & Malfois, M. (2013). *J. Appl. Cryst.,* **46**, 1289-1297.
